# The Genetic Architecture of Climatic Adaptation of Tropical Cattle

**DOI:** 10.1371/journal.pone.0113284

**Published:** 2014-11-24

**Authors:** Laercio R. Porto-Neto, Antonio Reverter, Kishore C. Prayaga, Eva K. F. Chan, David J. Johnston, Rachel J. Hawken, Geoffry Fordyce, Jose Fernando Garcia, Tad S. Sonstegard, Sunduimijid Bolormaa, Michael E. Goddard, Heather M. Burrow, John M. Henshall, Sigrid A. Lehnert, William Barendse

**Affiliations:** 1 CSIRO Food Futures Flagship, Queensland Bioscience Precinct, St. Lucia, QLD, Australia; 2 CSIRO Animal, Food and Health Science, Queensland Bioscience Precinct, St. Lucia, QLD, Australia; 3 Zoetis Incorporated, Parkville, VIC, Australia; 4 Garvan Institute of Medical Research, Darlinghurst, Sydney, NSW, Australia; 5 Animal Genetics and Breeding Unit, University of New England, Armidale, NSW, Australia; 6 Cobb-Vantress Inc., Siloam Springs, Arizona, United States of America; 7 Queensland Alliance for Agriculture and Food Innovation, The University of Queensland, St. Lucia, QLD, Australia; 8 Faculdade de Medicina Veterinaria de Araçatuba, (UNESP) Univ Estadual Paulista, Araçatuba, São Paulo, Brazil; 9 United States Department of Agriculture, Agricultural Research Service, Bovine Functional Genomics Laboratory, Beltsville, Maryland, United States of America; 10 Department of Primary Industries Victoria, Bundoora, VIC, Australia; University of North Carolina, Greensboro, United States of America

## Abstract

Adaptation of global food systems to climate change is essential to feed the world. Tropical cattle production, a mainstay of profitability for farmers in the developing world, is dominated by heat, lack of water, poor quality feedstuffs, parasites, and tropical diseases. In these systems European cattle suffer significant stock loss, and the cross breeding of taurine x indicine cattle is unpredictable due to the dilution of adaptation to heat and tropical diseases. We explored the genetic architecture of ten traits of tropical cattle production using genome wide association studies of 4,662 animals varying from 0% to 100% indicine. We show that nine of the ten have genetic architectures that include genes of major effect, and in one case, a single location that accounted for more than 71% of the genetic variation. One genetic region in particular had effects on parasite resistance, yearling weight, body condition score, coat colour and penile sheath score. This region, extending 20 Mb on BTA5, appeared to be under genetic selection possibly through maintenance of haplotypes by breeders. We found that the amount of genetic variation and the genetic correlations between traits did not depend upon the degree of indicine content in the animals. Climate change is expected to expand some conditions of the tropics to more temperate environments, which may impact negatively on global livestock health and production. Our results point to several important genes that have large effects on adaptation that could be introduced into more temperate cattle without detrimental effects on productivity.

## Introduction

Cattle are a key component of tropical rural sustainability, wealth, and food production for small holder farms, which are vital for global food security [1], [2]. Tropically adapted cattle breeds, usually of indicine ancestry, are less productive than European taurine cattle under favourable conditions [3], [4]. However, they are easier to maintain under conditions of low inputs, and remain productive under the onslaught of parasites, tropical diseases and high heat loads [5], [6], [7]. Nevertheless, lower innate productivity is a serious limitation to sustainable intensification of cattle productivity in the tropics.

The barriers to uptake of more productive cattle in tropical environments are lack of quality nutrition during the dry season, high temperature and humidity during the wet season, high water requirements, and devastating tropical parasites and infections, amongst others [8], [9], [10], [11]. Some breeders, particularly in developed countries, have responded by generating taurine-indicine composite cattle and then selecting the best animals to form new, composite breeds for particular environments or production systems.

Indicine and European taurine cattle differ substantially in many respects, not just in their productive potential in tropical environments, but also in traits such as temperament and appearance. First generation taurine x indicine crossbred cattle show heterosis including resistance to parasites and increased productivity, allowing them to outperform either taurine or indicine purebred cattle in tropical production systems [12], [13]. However, the causes of the decline of adaptation and productivity in subsequent generations may depend on the nature of the heterosis. This may be caused in part either 1) by genes of large, dominant effect that are near to being fixed for opposite alleles in the two subspecies, or 2) due to epistatic or pleiotropic interactions between genes within one subspecies that are then upset by new genetic combinations generated in the F2 or subsequent generations, or 3) due to recessive deleterious mutations in each group that are diluted in the first generation but through segregation emerge in the second generation, or 4) due to overdominance at a large number of loci, in which individuals in the F1 generation would on average have more loci with overdominant effects than individuals in the F2 generation [14], [15].

There has been anecdotal observation that some F2 crossbred cattle either perform substantially less favourably than F1 cattle, or seem to inherit the weak points of both parental breeds, which might suggest genes of large effect. However, evidence for genes of large effect in genome wide analyses has so far been lacking. Previous studies of crossbred animals have focussed on meat quality and efficiency of production in feedlots, and so far, only meat tenderness and reproductive performance have shown any evidence of genes of moderate effect [16], [17].

We have used a large sample of crossbred cattle of two types in genome wide association studies (GWAS) to analyse 10 traits that are important in extensive tropical environments. Such traits include overall productivity and yearling weight, heat and parasite tolerance, and temperament. So far, GWAS of parasite resistance traits have been performed with small sample sizes and have not identified any genes of large effect [18]. Here we show that contrary to the general expectation of a large number of genes each of infinitesimally small effect, that 9 of the 10 traits were affected by genes of moderate to large effect size. This suggests that for tropical productivity, the genetic differences between indicine and European taurine cattle includes the segregation of genes of large effect.

## Results and Discussion

**Figure 1:**
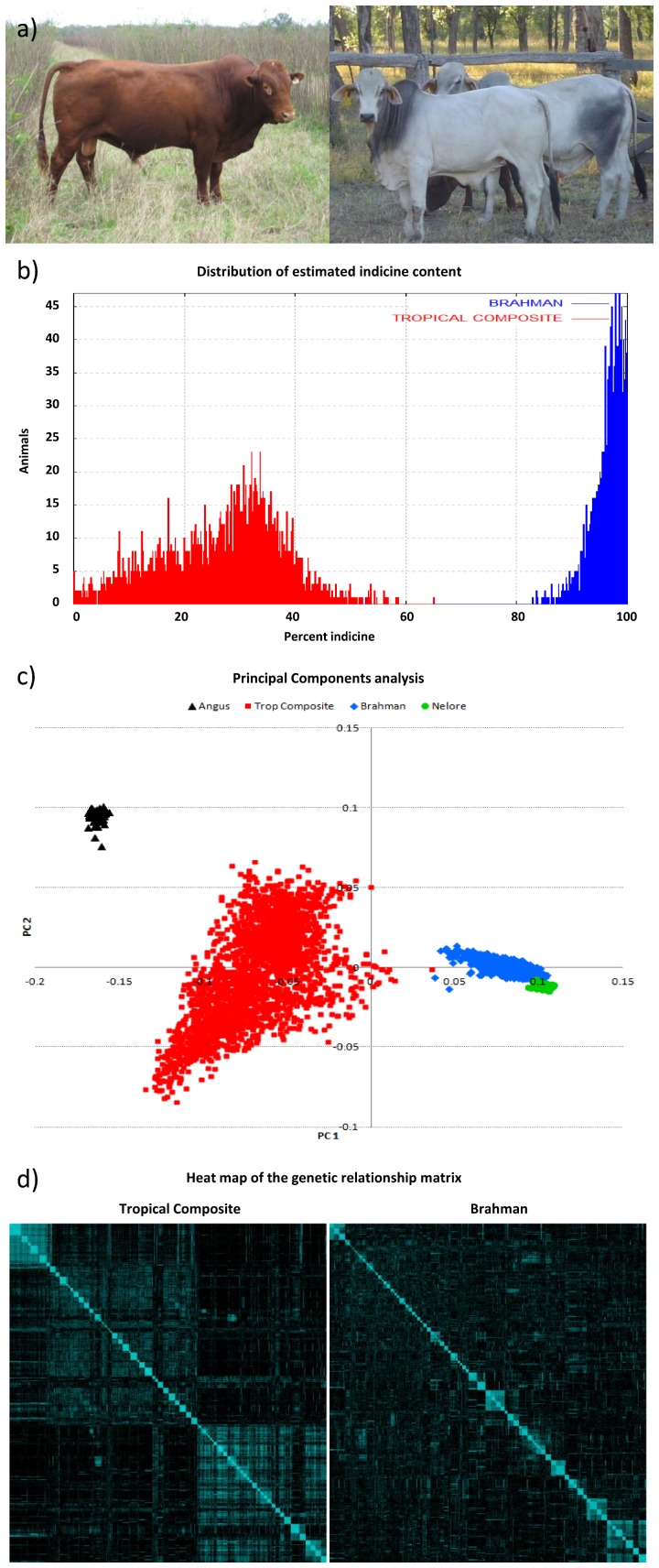
Cattle genetic differences: (a) a Tropical Composite (left) and a Brahman bull (right). (b) Distribution of indicine% across the two samples showing a mode at ∼30% for Tropical Composite (red) and ∼95% for Brahman (blue) cattle. (c) Multi-dimensional scaling plot showing a clustering of the Brahman (blue) and Tropical Composite (red) cattle relative to the reference samples of Angus (black) and Nelore (green). (d) Genetic relationship matrices based on genotype similarity among individuals for Tropical Composite (left) and Brahman (right) samples where the diagonal blocks show paternal half-sib families.

To determine whether there are genes of large effect segregating in taurine x indicine composite cattle we performed a genetic analysis of ten traits that are relevant for improved performance in tropical production systems (Table 1, Table S1). Here we consider the SNP to have a large size of effect if it accounted for more than 5% of the genetic variance, which translates to approximately 0.3 S.D. in allele substitution effect, due to the overall rarity of such genetic effects when measured in large sample sizes [16], [19], [20], [21]. We performed a genome wide association study (GWAS) using 4,662 pedigreed cattle and high density bovine single nucleotide polymorphism (SNP) arrays. There were 2,112 animals of the Brahman breed, which is largely of indicine origin [22] and 2,550 cattle of a set of composite cattle with varying amounts of European and African taurine and indicine ancestry, all raised in tropical environments (Figure 1A). We also made use of genotypic datasets of 81 Angus (taurine) and 91 Nelore (indicine) cattle from previous experiments including the Bovine HapMap [23], [24], [25] to quantify the population structure and breed composition of our samples (Figure 1B, C). The Brahman sample showed small differences to the Nelore sample in a multi-dimensional scaling analysis of the genotypes. In comparison, the Tropical Composites showed substantial variation not only between the taurine and indicine extreme captured by the first component, but also substantial variation captured by the second component, which may be due to African ancestry of the animals descended from the Belmont Red [26], Africander, and Senepol breeds.

**Table 1:** Adaptation phenotypes in Brahman and Tropical Composite cattle.

		FT	TEMP	EPG	SHEATH	COLOUR	FLY	TICK	COAT	COND	YWT
Brahman	N	2,112	1,183	996	2,111	2,093	983	675	1,891	2,112	2,110
	Mean	152.75	39.28	300.95	4.75	3.28	1.07	0.67	5.08	7.32	227.7
	SD	60.87	0.59	250.04	1.15	0.83	0.71	0.74	1.42	0.95	34.31
	SNP	1,870	1,086	1,772	5,766	6,154	1,851	1,045	3,026	4,683	3,751
	FDR	0.39	0.67	0.41	0.13	0.12	0.39	0.70	0.24	0.15	0.19
Tropical Composite	N	2,533	1,782	1,523	2,533	2,531	1,062	229	2,550	2,550	2,542
	Mean	141.16	39.01	318.38	7.39	3.80	0.74	2.52	6.62	7.00	247.07
	SD	48.76	0.83	305.33	1.44	0.85	0.51	0.93	2.20	0.83	44.03
	SNP	3,480	856	2,461	14,269	9,206	1,942	1,417	8,073	7,371	14,000
	FDR	0.21	0.85	0.30	0.05	0.08	0.37	0.51	0.09	0.10	0.05

Summary statistics (number of records (N), mean and standard deviation (SD)), effect of percentage of indicine (indicine%), heritability estimates (h^2^) and associated SNP (from a total of 729,068) and false discovery rate (FDR) at P<0.001. FT flight time (sec), TEMP rectal temperature (C), EPG worm eggs per gram of faeces, SHEATH pendulousness of the penile sheath (score), COLOUR coat colour (score), FLY lesions due to biting by buffalo flies (score), TICK tick score, COAT coat score, COND body condition score, YWT yearling weight (kg). Trait definitions and measurement procedure contained in Table S1.

Trait measurements included 1) measures of coat colour, score (length), and rectal temperatures (Table S1), which are relevant to heat tolerance, 2) temperament and sheath/navel score, which are relevant to the ease of care and management needed in tropical agriculture, 3) response to parasites (ticks, worms, flies), which relate to tropical diseases and parasites, and 4) yearling weight and body condition score, which measure the overall response of the animal to the conditions. The estimated heritabilities and genetic correlations were found to be similar in both samples (Table 2). Seven out of the 10 traits (Table 1, Table 2 and Table S1) showed moderate to high heritability (i.e., between 0.30 and 0.60), and the genetic correlation between traits in males and females was almost always close to one (Table S2). Two of the 10 traits were lowly heritable in both samples, the size of lesions due to fly infestation and rectal temperature. The first depends on *Stephanofilaria* infection from the fly salivary glands [27] and the second is a trait under negative feedback control. Only tick burden showed a moderate heritability in the Tropical Composite sample and a low heritability in the Brahman sample. This trait had the smallest number of observations, so the difference could be due to sampling. Previous estimates of heritabilities and genetic correlations for these traits have been estimated on smaller subsets of these data [28]. Overall, when comparing the 45 estimates of genetic correlations across the two samples, we did not find a significant difference (2 tailed t-test, *P* = 0.874) between them. This suggests that although the animals differed substantially in the amount of taurine or indicine ancestry, the amount of genetic variation and the additive genetic relationships between traits was similar in the two samples.

**Table 2:** Heritabilities (diagonal), genetic (top) and phenotypic (bottom) correlations.

BRAHMAN										
Trait	FT	TEMP	EPG	SHEATH	COLOUR	FLY	TICK	COAT	COND	YWT
**FT**	**0.44**	−0.375	0.088	0.131	−0.088	0.389	−0.121	0.180	0.075	0.030
**TEMP**	−0.176	**0.22**	0.080	0.097	0.201	−0.587	−0.456	−0.005	0.236	−0.209
**EPG**	0.042	−0.062	**0.39**	−0.058	−0.387	0.207	0.506	−0.002	−0.061	0.006
**SHEATH**	−0.015	−0.031	0.010	**0.51**	−0.017	−0.200	0.148	−0.092	0.293	0.032
**COLOUR**	−0.018	0.117	−0.132	−0.033	**0.54**	−0.072	−0.351	−0.183	0.063	−0.001
**FLY**	−0.036	−0.096	−0.017	−0.031	−0.049	**0.19**	0.044	0.349	−0.041	0.386
**TICK**	−0.013	−0.010	0.088	−0.010	−0.082	−0.055	**0.09**	−0.105	−0.055	−0.091
**COAT**	0.034	−0.016	0.028	−0.021	0.017	0.018	0.011	**0.41**	−0.231	−0.034
**COND**	0.001	0.021	−0.069	0.054	−0.008	−0.013	−0.004	−0.165	**0.45**	−0.093
**YWT**	0.033	−0.110	0.034	0.030	−0.021	0.036	0.089	−0.170	0.141	**0.38**

TROPICAL COMPOSITE										
Trait	FT	TEMP	EPG	SHEATH	COLOUR	FLY	TICK	COAT	COND	YWT
**FT**	**0.44**	−0.250	−0.058	0.103	−0.171	0.074	−0.283	−0.030	0.056	0.019
**TEMP**	−0.108	**0.14**	−0.076	−0.242	0.188	0.171	0.230	0.070	0.177	−0.169
**EPG**	−0.008	−0.043	**0.56**	0.046	−0.202	−0.248	0.376	−0.061	0.304	0.127
**SHEATH**	0.016	−0.043	0.092	**0.57**	−0.201	−0.193	−0.091	−0.058	0.314	−0.184
**COLOUR**	−0.044	0.021	−0.049	−0.119	**0.53**	0.193	0.621	−0.130	−0.030	−0.180
**FLY**	−0.090	−0.021	−0.025	−0.143	0.064	**0.27**	−0.404	−0.038	−0.085	−0.188
**TICK**	−0.025	0.069	0.088	−0.056	−0.143	0.077	**0.41**	0.065	−0.028	0.145
**COAT**	0.005	0.048	−0.001	−0.020	−0.051	−0.074	0.155	**0.53**	−0.074	−0.163
**COND**	0.007	0.036	0.042	0.089	0.011	−0.073	−0.073	−0.104	**0.31**	−0.129
**YWT**	−0.002	−0.077	−0.023	−0.130	−0.015	−0.021	−0.082	−0.135	0.126	**0.58**

To determine the role of indicine origin in the genetic architecture of these traits we quantified the extent of admixture percentage of each animal. Using genotypes for 228,268 SNP for the two reference samples of Angus and Nelore, we estimated the genomic proportion that can be attributed to indicine (indicine%) ancestry in each animal. When Hereford, Shorthorn and Gir were used as the reference samples they provided highly correlated estimates (r>0.91), demonstrating that the specific taurine or indicine breed was not important for quantifying indicine% in these samples (Figure S1). The mean indicine% of the Brahman sample was 96.7% while that of the Tropical Composite sample was 27.2% (Figure 1B). The estimated effect of indicine% was statistically significant (*P*<0.005) for 7 out of the 10 observed traits in the Tropical Composite sample, and for 4 traits in the Brahman sample (Table 1 and Table 3). In both samples, indicine% lightened coat colour, short coat score and caused more pendulous penile sheaths and increased weight and body condition score. In addition, indicine% reduced worm infestation and worsened temperament in the Tropical Composite sample. These changes were consistent with the general scientific and anecdotal description of indicine and European taurine cattle. Nevertheless, although these effects were statistically significant, the effect sizes were small. The observation of the empirical density having a clear mode at ∼30% of indicine% (Figure 1b) encouraged the exploration of the effect of indicine content separate for each group. The switching of size and/or sign in the estimated effect of indicine% in the two groups indicates a possible non-linearity of indicine gene effect in the transition from mostly taurine to mostly indicine. This was clearly the case for EPG.

**Table 3:** Estimated effect of indicine content (indicine%).

Trait	BRAHMAN (n = 2,112)			TROPICAL COMPOSITE								
				Full sample (n = 2,550)			Indicine% <30%^A^ (n = 1,375)			Indicine% ≥30%^A^ (n = 1,175)		
	Estimate	SE	P	Estimate	SE	P	Estimate	SE	P	Estimate	SE	P
**FT**	0.826	0.434	0.0571	−0.641	0.091	<.0001	−1.070	0.158	<.0001	0.320	0.252	0.2047
**TEMP**	−0.003	0.005	0.6118	−0.006	0.002	0.0004	−0.007	0.003	0.0128	−0.015	0.005	0.0015
**EPG**	3.912	2.212	0.0774	−5.180	0.761	<.0001	−9.717	1.398	<.0001	5.544	1.801	0.0021
**SHEATH**	−0.028	0.007	0.0001	−0.038	0.003	<.0001	−0.035	0.005	<.0001	−0.067	0.008	<.0001
**COLOUR**	−0.047	0.006	<.0001	−0.004	0.002	0.0399	−0.010	0.003	0.0013	0.021	0.005	<.0001
**FLY**	0.008	0.006	0.1810	−0.010	0.001	<.0001	−0.015	0.003	<.0001	−0.001	0.004	0.7449
**TICK**	−0.006	0.008	0.4438	0.014	0.008	0.0708	0.012	0.009	0.169	−0.381	0.207	0.0669
**COAT**	−0.022	0.010	0.0291	−0.037	0.004	<.0001	−0.001	0.007	0.8941	−0.073	0.011	<.0001
**COND**	0.016	0.005	0.0019	0.013	0.002	<.0001	0.017	0.003	<.0001	0.013	0.004	0.002
**YWT**	1.035	0.208	<.0001	0.135	0.068	0.0481	0.467	0.119	<.0001	−0.215	0.190	0.2574

A The observation of the empirical density having a clear mode at ∼30% of indicine% (Figure 1b) encouraged the exploration of the effect of indicine content separate for each group.

**Figure 2:**
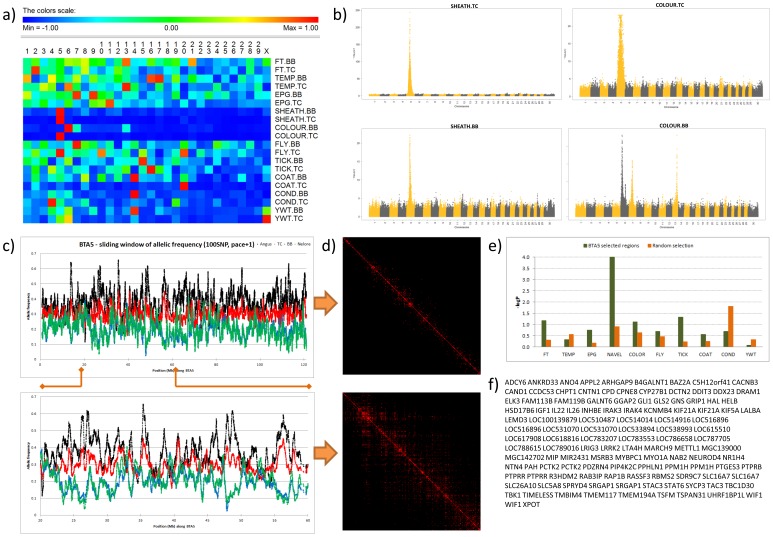
Genetic architecture of climatic adaptive traits: (a) Heat map of the number of significant SNP (*P*<0.0001) from few (blue) to many (red) across the 30 chromosomes for the ten traits in Tropical Composite and Brahman. (b) Manhattan plots of the significance (-log*P* on the y-axis) of each SNP in genome order (x-axis) for Sheath score (left panels) and Coat Colour (right panels) for Tropical Composite (upper panels) and Brahman (lower panel) cattle. Note the similarity on BTA5 for Sheath score in both samples but different genes for colour, (c) Average frequency of the forward allele for sliding windows of 100 consecutive SNP along BTA5 at 1 SNP pace for Brahman (blue) and Tropical Composite (red) cattle relative to the reference samples of Angus (black) and Nelore (green). The insert shows regions of divergent allele frequencies between taurine and indicine cattle on BTA5 from 20 to 60 Mb. (d) Heat map of LD (*r* ^2^) on BTA5 in the Tropical Composite sample, with red dots corresponding to *r* ^2^>0.1, showing LD blocks spanning several Mb. (e) The effect of BTA5 on Sheath score in Tropical Composite and Brahman cattle was not due to indicine% but to a major gene. 50 SNP were selected from 5 regions of BTA5 with divergent alleles in Angus and Nelore cattle. The average additive association (based on –log(*P*), y-axis) of these 50 SNP across the ten traits was calculated (green bars) and compared with the association of 50 SNP (orange bars) also with divergent alleles in Angus and Nelore cattle but randomly located to regions other than BTA5 or BTAX. A total of 10 random selections were chosen and the average plotted. (f) Genes close (< = 3 Kb) to SNP significantly associated (P<0.0001) with Sheath in both breeds.

In the GWAS, nine of the ten traits showed SNP of genome-wide significance (*P*<5×10E-8) (Table 4, Figure 2, Table S3, Table S4 and Figure S2 and Figure S3). Genetic regions identified by these SNP explained between 3.6% and 71.3% of the genetic variance (Table 4), with the proviso that these values were not shrunk through whole genome simultaneous estimation. Evidence of genetic control including genetic variants of large effect, as opposed to only due to genes of small effect, was observed for coat colour, coat score, penile sheath, yearling weight, and body condition (Figure 2A). While coat colour in cattle is known to be controlled by a small number of genes of large effect [29], the finding for other traits including penile sheath and yearling weight has not been demonstrated before. Some of the alleles affecting parasite resistance exceeded 5% of the genetic variance, larger than previous estimates based on smaller sample sizes [18].

**Table 4:** Locations of the SNP explaining the largest genetic variance for each trait within each breed of cattle^1^.

Trait	SNP	Location^2^	Distance to the closest candidate gene	Closest annotated candidate gene	Brahman		Tropical Composite	
					%Vg^3^	-log*P* ^4^	%Vg	-log*P*
Flight time (Temperament)	BovineHD0900013602	9:49377393	158842	GRIK2	3.48	6.7	0.02	0.2
	BovineHD0800005162	8:16543703	105237	SNORD35	0.05	0.3	3.58	8.4
Rectal temperature	BovineHD1700005935	17:20511693	90579	PCDH18	11.40	6.2	0.47	0.5
	BovineHD0600001261	6:4815627	72491	PRDM5	0.05	0.1	11.03	5.5
Worm Eggs per gram faeces	BovineHD2100019447	21:66320006	1436	CYP46A1	7.44	8.4	0.23	0.6
	BovineHD1100020520	11:71745768	0	BRE	6.34	2.8	4.19	10.1
Sheath score	BovineHD0500013322	5:46352065	18122	DYRK2	9.87	22.2	28.82	84.1
	BovineHD0500014002	5:48506281	57525	MSRB3	7.56	17.3	71.26	224.9
Coat colour	BovineHD1300018314	13:64153417	81228	ASIP	12.60	23.1	1.95	5.3
	Hapmap60668-rs29018280	5:57681031	0	DGKA	0.07	0.4	16.67	23.1
Fly lesions	BovineHD2200007206	22:24455446	598190	CNTN6	6.52	8.3	0.17	0.2
	BovineHD1000016441	10:55501172	3810	SNORA25	0.54	0.5	10.06	7.1
Tick score	BTA-24463-no-rs	13:22021716	6393	PLXDC2	10.06	9.6	0.97	1.3
	BovineHD0200039167	2:134264081	4115	U3	0.48	0.1	30.63	6.7
Coat score	BovineHD0800010960	8:36772960	0	PTPRD	4.62	7.4	0.38	1.5
	BovineHD2000009323	20:32481431	124618	FBXO4	0.01	0.1	15.22	39.2
Body Condition Score	BovineHD1400007259	14:25015640	6344	PLAG1	5.59	10.6	4.23	6.8
	BovineHD0400021316	4:77032763	62422	ADCY1	0.86	2.2	6.31	10.6
Yearling Weight	BovineHD4100004494	6:37534689	0	HERC3	5.66	8.6	1.75	5.1
	BovineHD1400007259	14:25015640	6344	PLAG1	3.06	5.1	4.44	13.1
	BovineHD0500013787	5:47724746	0	HELB	0.19	0.6	5.35	13.7

1 The estimated SNP effect is given for both breeds even though rarely a SNP explained a large proportion of the variance in both breeds. ^2^ SNP location, chromosome:position (bp). ^3^ Percent of the genetic variance explained by the SNP. ^4^ P-value for the genome-wide association.

Although the size of the genetic variance in each sample was similar for all traits, as measured by the estimated heritability, the regions of the genome with the largest effects were generally different between the two samples, except for sheath score. For example, SNP related to the genes *ASIP* and *PLAG1*, which had important effects on coat colour or body condition score in both samples, accounted for different amounts of genetic variance in each sample (Table 4). Animals of the two groups show significant differences in coat colour and structure, response to parasites, size and body shape (Figure 1A and Table 1), and so major genetic differences between them may be expected. Traits with low heritability showed few associations that were genome wide significant and little to no overlap between the two samples, suggesting that even larger sample sizes will be needed to analyse these complex traits in detail.

The most notable results were for penile sheath and coat colour in the Tropical Composite sample, in which almost the entire length of bovine chromosome (BTA) 5 was associated to both traits (Figure 2B). Even restricting the analysis to a SNP association value of –log*P*>20 resulted in more than 50 Mb of the chromosome associated to either trait, with a massive overlap of these BTA5 segments between traits. We took several approaches to determine if this was an artefact of analytical inputs. We found evidence of significant differences in allele frequency between Angus and Nelore in several regions of BTA5. However, other regions of the genome with such differences in allele frequency did not show effects on coat colour or penile sheath, so the allele frequency differences do not explain the observations (Figure 2C and E). Although there was evidence of long range linkage disequilibrium (LD) across most of BTA5 (Figure 2D) in this sample, and such LD might explain correlated –log*P* values over extended regions of a chromosome, BTA5 is not special compared to other chromosomes in this data set and indeed shows lower levels of LD between SNPs at different distances than the average of other autosomes (Dataset S1 taken from Porto-Neto and coworkers [30]). Regions of the genome containing *PLAG1* [31] and the *Slick* locus [32] gene also show broad regions of significance in this sample, with *PLAG1* on BTA14 showing –log*P*>8 over 4.4 Mb for yearling weight. The region containing the *Slick* gene showed –log*P*>20 over 28 Mb. In the Tropical Composite breeds studied here, the gene is likely to be derived from crossing with bulls of part Senepol ancestry, a known source of this trait. Since the colour and sheath score genes are unlikely to have a similar defined origin, this suggests that more than LD is involved. To determine whether these effects were due to recent crossbreeding, we excluded all animals of recent crossbred origin, restricting the analysis to 921 animals that have been closed to introgression for many generations and found the same broad extent of association. We examined the haplotype structure of BTA5 in this restricted sub-population of animals from both breed types and found that it contained significant haplotypes associated to both coat colour and penile sheath score extending over at least 10 Mb in size (Dataset S2). Moreover, in addition to these two traits, this region is also associated to yearling weight, body condition score, and parasite resistance (Table 5, Table S5) and female reproductive success in previous studies [17]. Given this combination of traits in one genomic region, we speculate that breeders have maintained haplotypes of genes for coat colour and penile sheath conformation because they are linked to parasite resistance and productivity in tropical environments, possibly because these traits are signatures of *Bos indicus* ancestry. However, we found that genetic variants leading to more pendulous penile sheaths, derived from Brahman breed ancestry, were associated with reduced resistance to parasites, lower yearling weight and better body condition scores in these samples. One would have expected improved resistance to parasites from the Brahman alleles.

**Table 5:** Studentized effect (estimated effect divided by its standard error) of seven selected pleiotropic SNP and its colosest genes across five adaptation phenotypes in Brahman (BB) and Tropical Composite (TC) cattle.

SNPid and position (BTA:bp)	Gene	Distance to gene (bp)	Breed	Phenotype				
				SHEATH	COLOUR	COAT	COND	YWT
BovineHD0500012231 (5: 42818597)	PTPRR	19000	BB	−5.511	−0.511	3.894	−1.273	0.409
			TC	−18.522	5.753	−2.063	−3.827	3.282
BovineHD0500013895 (5: 48069099)	HMGA2	0	BB	−10.361	−0.286	1.780	−3.964	0.018
			TC	−41.397	6.291	−0.914	−5.244	8.146
BovineHD0500016019 (5: 56371072)	INHBC	20629	BB	−8.204	1.044	1.515	−3.485	−0.400
			TC	−26.961	10.142	0.330	−4.965	4.173
BovineHD0600005016 (6: 18348426)	LEF1	0	BB	0.662	−20.531	0.228	−0.735	0.083
			TC	2.479	−2.741	−0.915	0.002	−4.335
BovineHD1300018328 (13: 64228423)	ASIP^1^	10801	BB	−0.139	11.329	−0.653	0.199	−0.767
			TC	−0.647	4.156	0.797	−3.711	−0.529
BovineHD1400007257 (14: 25009960)	PLAG1	664	BB	−0.690	0.692	−4.686	8.195	−5.778
			TC	1.165	0.721	0.002	6.303	−4.999
BovineHD2000008759 (20: 29858632)	MRPS30	213583	BB	−0.836	0.868	1.502	0.838	−3.941
			TC	2.213	−0.951	10.736	0.618	−0.004

1 Candidate gene, the closest gene is SNORA73 at 624 bp.

To determine whether any of the linked effects may instead be pleiotropic in origin, in particular effects that might be antagonistic, we evaluated the effect of each SNP on all traits using the summation of standardized effects (Table 5 and Table S5). Genes on BTA5, 14 and 20 in particular, showed antagonistic effects for body condition and yearling weight, that is, the favourable allele for body condition was the unfavourable allele for yearling weight rate, suggesting an underlying genetic antagonism consistent with the slightly negative genetic correlation between these traits in both samples. Furthermore, some of these same favourable alleles for body condition or yearling weight have unfavourable effects on several of the parasite resistance traits, again consistent with the slightly negative genetic correlations between these traits. This suggests that selection for these genes of large effect on weight or body condition will come with a trade-off of reduced parasite resistance, which will need to be accommodated by selection for parasite resistance using the rest of the genome. Two of the genes that have antagonistic effects on more than one trait have been identified before, due to their large effects on the visible phenotype, viz. *PLAG1* on BTA14 associated with hip height and female reproductive performance [31], [33], and the *Slick* gene on BTA20 associated with coat score, ability to sweat, rectal temperatures [32], and hence ability to be active and feed during the heat of the day in the tropics. In this study we also identified an extended genetic region centred around the *MSRB3* gene on BTA5 as having large effects on several traits.

To identify the genes that underpin effects on yearling weight and body condition, we combined information on location of significant SNP effects and gene expression profiles of Tropical Composite cattle under tropical dry season conditions, where typical reductions in weight and body condition occur. SNPs that were significantly associated with yearling weight or body condition score in both samples identified a list of genes that had been examined for gene-expression differences using muscle biopsies before and after a period of low food availability for the Belmont Red breed [34], [35]. Of the 175 genes associated with significant SNP for condition score, 63 showed significant differences in gene expression before and after the period of low food availability, while 52 of 213 genes for yearling weight showed differences in gene expression (Table 6). Of great relevance for periods of undernutrition, some of these genes were linked to energy metabolism (*LDHA*, *IGFBP4*, *ATP7A*, *STEAP2* and *MON2*), muscle metabolism (*MYH2*, *ASPH* and *APOBEC2*), fertility (*INHBC*) and the immune system (*LYN*, *CHD7*, *ADORA2B*, *IRAK3*, *CORO2A* and *TOX*). A large number of these genes were associated with the region containing *MSRB3* on BTA5 and *PLAG1* on BTA14 as noted above. This suggests not only that some of the genetic variation affects the phenotype through differences in gene expression, that clusters of genes affecting these traits are co-ordinately expressed, but also that genetic variation in physiologically relevant gene networks are deployed by the animal to adapt to tropical conditions.

**Table 6:** Selected positional-candidate genes (P<0.01 both breeds) and its different-expression (P<0.05) in muscle before and after undernutrition period*.

BTA	Mb	Gene id	DE	Ref trait	COND						YWT					
					BRM			COMP			BRM			COMP		
					a	%Vg	LogP	a	%Vg	LogP	a	%Vg	LogP	a	%Vg	LogP
2	65	LYPD1	−3.308	C	0.064	1.14	2.426	0.052	1.23	2.320	0.024	0.00	0.013	0.298	0.01	0.153
4	74	STEAP2	−5.063	C	0.077	1.49	3.289	0.078	1.88	3.461	−0.444	0.06	0.265	−1.272	0.16	0.833
4	83	AMPH	−3.994	C	−0.086	2.10	4.247	−0.064	1.63	2.959	−0.385	0.05	0.235	0.812	0.08	0.516
4	116	DPP6	−3.003	C	−0.057	0.91	2.088	−0.053	0.99	2.008	−0.675	0.14	0.460	0.629	0.04	0.349
5	51	MON2	1.888	Y	0.03	0.25	0.742	−0.082	1.42	2.648	1.928	1.15	2.014	4.996	1.66	5.285
5	56	INHBC	−1.756	C	−0.131	0.95	2.365	−0.053	1.20	2.110	−1.566	0.15	0.524	1.297	0.23	1.000
8	63	CORO2A	3.298	C	0.113	1.44	3.110	0.052	1.19	2.024	2.388	0.71	1.498	1.480	0.30	1.138
14	24	LYN	3.126	C, Y	−0.141	5.66	10.440	−0.106	4.33	6.983	3.270	3.39	5.513	5.647	3.88	11.551
14	24	TGS1	1.597	C, Y	0.146	6.04	11.078	0.088	2.66	4.592	−2.406	1.83	3.200	−4.996	2.71	8.457
14	24	TMEM68	1.619	C, Y	−0.146	6.04	11.078	−0.094	3.23	5.419	2.406	1.83	3.200	5.362	3.32	10.121
14	25	PENK	−1.999	C, Y	−0.134	5.01	9.307	−0.076	1.67	3.071	2.950	2.71	4.500	4.322	1.71	5.514
14	25	PLAG1	2.067	C, Y	0.14	5.56	10.100	0.117	4.24	6.618	−3.387	3.63	5.785	−3.931	1.51	4.654
14	26	NSMAF	1.628	C, Y	0.149	6.11	11.202	0.068	1.78	2.991	−1.885	1.09	2.085	−3.043	1.13	3.502
14	26	TOX	−2.119	C, Y	0.152	6.34	11.652	0.084	2.67	4.400	−1.918	1.13	2.135	−2.570	0.79	2.690
14	27	RAB2A	1.956	C	−0.132	4.90	8.830	−0.041	0.76	1.550	2.907	2.65	4.264	1.981	0.56	2.064
14	28	ASPH	−1.613	C, Y	−0.131	4.81	8.870	−0.065	1.62	2.754	2.850	2.54	4.186	3.566	1.54	4.580
14	28	CHD7	3.484	C, Y	−0.141	5.64	10.177	−0.078	2.17	3.491	2.996	2.84	4.572	3.205	1.16	3.564
14	40	CRISPLD1	−6.004	Y	0.045	0.54	1.371	0.001	0.00	0.000	−1.943	1.12	2.120	−2.442	0.62	2.192
15	39	SPON1	−8.754	Y	−0.036	0.12	0.489	0.069	1.57	2.836	−3.683	1.37	2.640	−2.583	0.69	2.502
19	30	MYH2	−2.159	C	0.059	0.99	2.119	0.082	2.35	4.269	−0.749	0.18	0.523	1.026	0.12	0.682
19	41	IGFBP4	−1.848	C	0.13	1.14	2.407	0.078	2.56	4.058	2.379	0.42	0.967	1.680	0.37	1.471
30	66	LDHA	−4.816	Y	−0.024	0.14	0.712	0.033	0.35	1.273	1.611	0.70	2.127	3.496	1.23	6.642
30	79	ATP7A	1.969	Y	0.007	0.01	0.118	−0.045	0.77	2.388	−1.970	0.70	2.053	−2.544	0.78	4.223

*BRM – Brahman; COMP – Tropical Composite; DE – fold change in gene-expression; Ref trait – trait for which the gene was significantly associated: C – COND, Y – YWT; a – estimated SNP effect; LogP BRM and LogP COMP – -log(p-value) in Brahman and Tropical Composite. If a gene had more than one SNP associated to the trait, the SNP with lower p-value combined in both breeds was selected to represent the gene. REML estimates of genetic variance: COND BRM  = 0.175, COND COMP  = 0.110, YWT BRM 157.30, YWT COMP 348.14.

Climate change is expected to expand conditions of the tropics to more temperate environments, which may impact negatively on global cattle production unless steps are taken to improve cattle adaptation and productivity. Not only will temperate regions become warmer but climatic variability will increase and the range of devastating tropical and subtropical parasites will extend into temperate regions [36]. Due to the limits on arable land [37] and increased requirement by other industries for grain and other feedstuffs currently fed to livestock, cattle production systems must become more efficient [1]. Cattle will be expected to improve productivity and increase adaptation to heat, diseases, and poor quality feed on marginal land, to add to the total food available. Our results point to several important genomic regions that have large effects on adaptation without compromising productivity. They also show that the genetic architecture for adaptation to the tropics includes alternative alleles of large effect rather than co-adaptation of the genome that breaks down during crossbreeding between the subspecies. This suggests that a focus on these genes would rapidly improve the predictability of crossbreeding of taurine and indicine cattle. Finally, sustainably intensifying livestock systems in tropical regions may need genetic safeguards to ensure that productivity is raised while also adapting to climate change. The results of this study are a necessary step towards achieving that goal.

## Materials and Methods

### Animals

Animal Care and Use Committee approval was not obtained for this study because no new animals were handled in this experiment. The experiment was performed on trait records and DNA samples that had been collected previously. The resource population used in this study had been established by the Cooperative Research Centre for Beef Genetic Technologies (Beef CRC) to understand the genetic links between adaptability and components of herd profitability in northern Australia [38]. DNA had been extracted from venous blood samples from each animal using Qiagen kits as previous described [39].

### Tropical adaptation-related traits

Tropical cattle are exposed to several environmental stressors such as hot and humid conditions, restricted water supply, periodic challenges from exo- and endo-parasites and the diseases they transmit, and seasonal under-nutrition due to low protein availability and feed digestibility. In this study, we examined 10 traits (Table S1): flight time (FT), rectal temperature (TEMP), endoparasite eggs per gram measured in faeces (EPG), penile sheath score (SHEATH) expressed as the correlated trait navel score in females, coat colour (COLOUR), buffalo fly lesions (FLY), tick infestation (TICK), coat score (COAT), condition score (COND) and yearling weight (YWT) [28]. While some of the traits are obviously related to tropical conditions, such as direct measures of heat or parasite resistance, all traits have relevance to tropical agriculture. Flight time is a measure of temperament, how excitable or nervous the animal is when exposed to humans, which is relevant because in much Australian tropical agriculture the animals are not regularly exposed to humans and hence can react and cause injury to themselves or their handlers. Coat colour and length are directly related to heat absorption and radiation and affect heat tolerance and water usage. Sheath or navel score are related not only to the performance of the bull, as animals with pendulous sheaths are more likely to be injured, but it is thought by many cattle breeders that pendulous sheaths, navels, dewlaps and long ears help to radiate heat, although this has not been proven. To improve consistency between subjective scores and across different sites, these scores were taken by only four trained individuals. Repeated measures on these tropical adaptive traits were collected at various post-weaning ages with specific measures defined based on the biological significance of the age at which the measurement was taken and to maximise the number of records available for analyses across all cohorts.

### Trait heritability and correlations

The estimates of genetic and phenotypic variances, heritability of each trait and phenotypic and genetic correlations between traits were derived from bivariate animal model analyses of Brahman and Tropical Composite performed independently using VCE 6.0.2 (ftp://ftp.tzv.fal.de/pub/vce6/). Genetic parameter estimates were calculated based on the genotyped animals and their pedigree consisting of 5 generations of ancestors. Analytical models included the fixed effects of contemporary group (combination of sex, year and location), age of dam and estimated indicine percent of the individual as covariate; as well as animal as random additive effect and the random residual component. Age of dam was found to be statistically significant as a covariate. Animals that are older, in a tropical area, will have had a longer time to adjust to heat, parasites, diseases, poor fodder, than younger animals, who would also produce less milk than older mothers. These differences will include the development of antibodies, some of which will be passed on to their offspring. Therefore, dams that are older will likely be more successful in raising offspring, and indeed, we see strong significant effects for this covariate. For YWT the additional covariate of AGE12 was also fitted into the model. AGE12 is the average age of the animal at days in which body weight was recorded (from all the weight measurements taken between 300 and 420 days of age) and so is a proxy for yearling age. The genetic variance and correlation for each trait was also calculated between males and females. Large differences in these statistics might be due to differential responses of the animals to the environment or because the traits may have different genetic architectures in males and females. To compare the correlation coefficients found for these traits between Brahman and Tropical Composite animals, the differences between correlation coefficients for each pair of traits between Brahman and Tropical Composite were compared using a paired t-test. This generated a single test for the 45 comparisons, rather than comparison the differences of the correlation coefficients to the standard errors and then applying a False Discovery Rate model for the number of tests performed.

### Genotypes

For the present study, we used 2,112 Brahman and 2,550 Tropical Composite cattle from the resource population genotyped using either the BovineSNP50 [40] or the BovineHD BeadChip (Illumina Inc., San Diego, CA) that includes more than 770,000 SNP.

All SNP had been mapped to the UMD build assembled by the University of Maryland [41] using the updated version 3.1 of the genome (available from Genbank accession DAAA00000000.2 and at http://www.cbcb.umd.edu/research/bos_taurus_assembly.shtml). Animals genotyped using the lower density array had their genotypes imputed to higher-density based on the genotypes of relatives, consisting of 589 Tropical Composite and 304 Brahman animals including all available sires, that had been genotyped using the BovineHD BeadChip. The imputation was performed within breed using as reference 519 Brahman and 351 Tropical Composites genotypes using the BovineHD (Illumina) and 30 iterations of BEAGLE [42], which resulted in a final dataset of 729,068 SNP genotypes per individual as reported in Bolormaa et al. [24]. For the estimation of indicine content, the full genotype dataset was filtered by linkage disequilibrium (LD) to reduce redundant information and optimise computation utilization. The LD filter was applied using PLINK v1.07 [43] in a sliding window consisting of 50 adjacent SNP, and if r^2^>0.5 was detected between a pair of SNP one of the SNP was removed, and then LD for the window was re-calculated. Once no more pairs of SNP had r^2^>0.5 the window moved 10 SNP along the chromosome. This procedure yielded a dataset containing 227,085 SNP. For SNP association analyses, the SNP were consistently encoded for all traits as AA, AB and BB using the TOP/BOT encoding of Illumina (http://http://res.illumina.com/documents/products/technotes/technote_topbot.pdf checked 30 July 2014) and then converted into numerical values of 0, 1, and 2 B alleles.

### Identification of population sub-structure and indicine content

We used PLINK v1.07 to calculate multi-dimensional scaling (MDS) and genetic relationship matrices based on the genotypes to quantify the sample substructure for both the full dataset as well as the LD filtered dataset. The Angus and Nelore data were used as representatives of pure European taurine and pure indicine animals respectively. We note that indicine percent lines up with the first principal component of a principal component analysis, whereas African ancestry lines up with the second principal component, where the full diversity of cattle is included in the same analysis [44].

The Tropical Composite sample consisted of beef industry lineages formed using indicine, Sanga and taurine cattle [38], and included animals with African taurine and no reported indicine ancestry, such as some sectors of the Belmont Red breed. The Brahman breed in Australia started in the 19^th^ century from various Indian cattle including animals from the Melbourne Zoo, upgraded by American Brahman and the Indu-Brazilian breeds so also includes a small proportion of taurine ancestors [22], [45], [46]. It therefore includes breeds such as the Kankrej (Guzerat), the Ongole (Nelore), and the Gir (Gyr). To estimate the amount of taurine and indicine content in the Tropical Composite and Brahman animals, HD genotypes for 81 Angus (Beef CRC) and 91 Nelore cattle were used as a reference. Estimates of indicine ancestry were also obtained using 55 Hereford, 54 Shorthorn, 44 Angus and 50 Gir. Some Angus, and the Hereford and Shorthorn samples were obtained from the Beef CRC database and some Angus, and the Nelore and Gir samples were obtained from the Bovine Hapmap [23], with some Nelore and Gir animals sampled from Brazil [25]. Ancestry was estimated using Admixture software [47] on the LD filtered dataset (∼228 K SNP) under supervised mode. Most of these Tropical Composite cattle were descended from the Hereford or Shorthorn breeds as taurine ancestors. Thereafter to evaluate the potential impact of breed on the indicine estimates, these were also obtained using different combinations of Hereford, Shorthorn and Gir. The correlation between different estimates of indicine content were>0.91 for these comparisons.

To estimate the effect of indicine percent on adaptability-related traits a linear model was fitted using SAS (SAS Inst., Cary, NC). The covariates used for the estimation of genetic parameters were used as fixed effects. Given the number of comparisons, the type I significance threshold was Bonferroni adjusted to α = 0.005.

### Genome-wide association studies

Genome-wide association studies (GWAS) were performed separately within each breed and for each of the ten traits using the final dataset with 729,068 SNP. The GWAS were performed one SNP at a time using the same univariate linear mixed models stated above, which included the fixed effects of contemporary group (combination of year and location), age of dam and estimated indicine percent of the individual as covariate; as well as animal as random additive effect and the random residual component and the SNP genotype (recoded as 0, 1 or 2) as an additional linear covariate. Solutions for the SNP effects and associated P-values were obtained using Qxpak5 [48]. As the coefficient for each SNP is provided as a signed number, the signs of significant coefficients of the same SNP for different traits can be compared to determine whether the SNP shows effects in the same or opposite directions between two traits. To determine whether fitting indicine percent substantially changed the outputted results, we compared GWAS output with and without indicine percent in the Brahman sample and found that the allele effects across traits had an average correlation of 0.97 (Table S6).

### False discovery rate

Following Bolormaa et al. [24], the false discovery rate was calculated as 
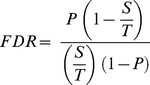



where *P* is the P-value tested (e.g., 0.0001), *S* is the number of SNP significant at the P-value tested and *T* is the total number of SNP tested (i.e. 729,068).

### Percentage of genetic variance explained by each SNP

The percentage of the genetic variance explained by the *i*-th SNP was estimated for each sample separately according to the following formula:
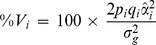



where *p_i_* and *q_i_* are the allele frequencies for the *i-*th SNP calculated for each breed, 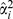 is the estimated additive effect of the *i*-th SNP on the trait under analysis, 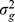 is the REML estimate of the (poly-)genetic variance for the trait, and 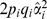 is the estimated additive variance of the *i*-th SNP in the absence of dominance [49].

### Test for pleiotropy

In both the Brahman and the Tropical Composite sample, the effects of 729,068 SNPs estimated from the GWAS were divided by their associated standard errors to obtain a t-value corresponding to the studentized SNP effects [50]. A multi-trait test of the i-th SNP was performed by storing its studentized effects across the 10 traits in the 10×1 vector **t_i_**. Then, the quadratic form **t_i_**'**V^-1^t_i_**, where **V** is the correlation matrix among the SNP effects, is distributed approximately as a chi-squared with 10 df under the null hypothesis that the SNP does not affect any of the traits. The correlation matrix **V** was calculated using the estimated SNP effects across the 729,068 SNPs. Custom code in FORTRAN 95 was developed to perform these operations. SNP were taken as having a significant pleiotropic effect when the quadratic form **t' V^-1^ t** exceeded 29.588 (i.e., P<0.001 from a Chi-squared distribution with 10 degrees of freedom). Here we are looking for pleiotropic effects for SNP that had already been demonstrated to be significantly associated to the traits of interest after correction for multiple testing, so no further correction for multiple testing was required.

### Gene-expression of positional candidate genes in muscle biopsies under environment stress

Positional candidate genes were defined as those genes within 3,000 bp of a SNP significantly associated (P<0.01) to either body condition score or yearling weight, and in which the same allele was favourably associated with the trait in both samples. To further explore those associated genes and link their responses to a potential environmental stressor, we evaluated their expression using data from a previously described cattle undernutrition experiment [34], [35]. The artificial feed reduction was to mimic natural drought conditions when food is of low protein composition and poor digestibility and cattle lose weight. Muscle biopsies from 12 Tropical Composite animals were collected before and after an undernutrition period of 114 days. Gene-expression was assessed using microarrays (ViaLactia Bioscience in collaboration with Agilent) that included more than 21,000 probes representing around 19,500 protein-coding genes. Genes that had significant (P<0.05) expression change in the compared period were selected and matched to those candidate genes derived from the GWAS.

### Estimated effect of the number of “taurine alleles” on adaptation-related traits

There are many SNP across the cattle genome that have one allele fixed or near fixation in indicine cattle while the alternative allele is fixed or near fixation in taurine animals. If a SNP had an allele with frequency>0.95 in Angus (taurine) and <0.05 in Nelore (indicine) animals, that allele was called the “taurine” allele and the alternative allele the “indicine” allele and labelled as a highly divergent SNP.

We noted a QTL of large effect on BTA5. To determine whether highly divergent SNP *per se* were associated to trait values or were contributing to the apparent size of the QTL effect, we collected genotypes for 50 highly divergent SNP distributed across 5 regions of BTA5. As a control, we collected 10 random selections each of genotypes for 50 highly divergent SNP for all sectors of the genome except BTA5 and BTAX. Then, the “indicine” and “taurine” alleles were identified and the number of “taurine” alleles was summed for each animal. To estimate the effect of the sum of “taurine” alleles on adaptability-related traits a linear model was fitted using SAS (SAS Inst., Cary, NC). The model contained the same fixed effects as the model for estimated indicine percent.

## Supporting Information

Figure S1 **Correlation of breed composition (indicine%) estimated using different sets of animals as reference populations.**
(DOCX)

Figure S2 **Manhattan plots of genome wide association for FT, TEMP, EPG, SHEATH, and COLOUR.**
(DOCX)

Figure S3 **Manhattan plots of genome wide association for FLY, TICK, COAT, COND, and YWT.**
(DOCX)

Table S1 **Trait definitions and abbreviations.**
(DOCX)

Table S2 **Bi-variate analysis for same trait in different sexes: Additive genetic variance (Va), heritability (h^2^) and genetic correlation (rg).**
(DOCX)

Table S3 **Number of significant SNP from GWAS at different levels of stringency for each trait and false discovery rate between brackets.**
(DOCX)

Table S4 **Summary table of SNP association: overlap regions between Brahman and Tropical Composite (P<0.0001 both breeds).**
(DOCX)

Table S5 **Selected Pleiotropy results: Studentized SNP effects across the 10 phenotypes for candidate SNP and its closest gene.** Highlighted in yellow are effects> 2.0 in absolute value.
(DOCX)

Table S6 **Correlation between SNP additive effects (n = 729,068), and statistical model R^2^ estimated in the Brahman population with and without fitting the covariate “% indicine” in the model.**
(DOCX)

Dataset S1 **Linkage Disequilibrium decay on BTA5 compared to the** ***other*** **autosomes.**
(XLSX)

Dataset S2 **Haplotype distribution in Tropical Composite animals for BTA5 affecting NAVEL and COLOUR.**
(XLSX)
