# Supplementary material for: The Genetic Architecture of Climatic Adaptation of Tropical Cattle
Source: PLoS One. 2014 Nov 24;9(11):e113284. doi: 10.1371/journal.pone.0113284 (PMC4242650; doi:10.1371/journal.pone.0113284)
Supplement: Figure S1 — Correlation of breed composition (indicine%) estimated using different sets of animals as reference populations. (DOCX) [file pone.0113284.s001.docx]

Figure S1. Correlation of breed composition (indicine%) estimated using different sets of animals as reference populations.

**
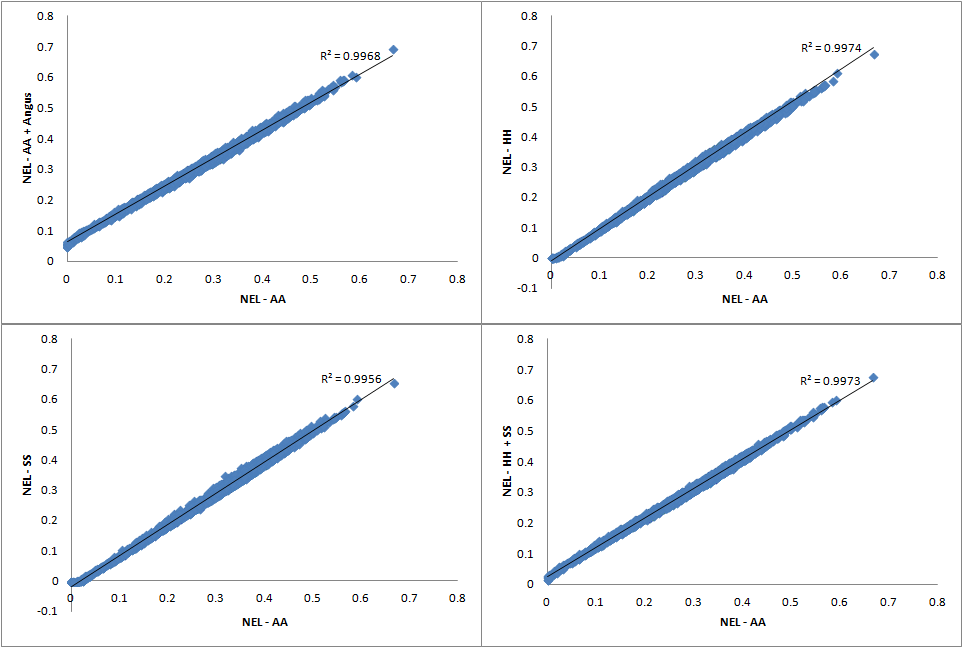
**
