# Supplementary figures and images for: The Genetic Architecture of Climatic Adaptation of Tropical Cattle
Source: PLoS One. 2014 Nov 24;9(11):e113284. doi: 10.1371/journal.pone.0113284 (PMC4242650; doi:10.1371/journal.pone.0113284)

Figure S2. Manhattan plots of genome wide association for FT, TEMP, EPG, SHEATH, and COLOUR.

**
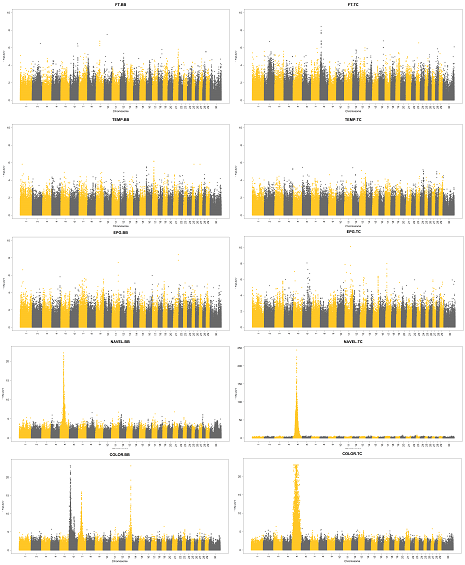
**

Supplement: Figure S2 — Manhattan plots of genome wide association for FT, TEMP, EPG, SHEATH, and COLOUR. (DOCX) [file pone.0113284.s002.docx]

Figure S3. Manhattan plots of genome wide association for FLY, TICK, COAT, COND, and YWT.

**
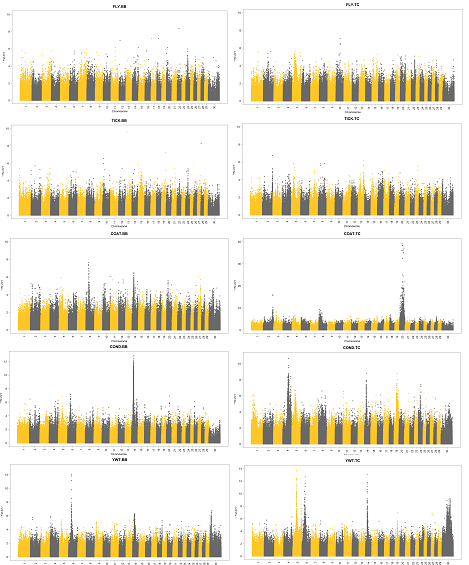
**

Supplement: Figure S3 — Manhattan plots of genome wide association for FLY, TICK, COAT, COND, and YWT. (DOCX) [file pone.0113284.s003.docx]
