# Supplementary material for: The Genetic Architecture of Climatic Adaptation of Tropical Cattle
Source: PLoS One. 2014 Nov 24;9(11):e113284. doi: 10.1371/journal.pone.0113284 (PMC4242650; doi:10.1371/journal.pone.0113284)
Supplement: Table S1 — Trait definitions and abbreviations. (DOCX) [file pone.0113284.s004.docx]

Table S1. Trait definitions and abbreviations.

| Trait | Code | Description |
| --- | --- | --- |
| Flight Time (s*100) | FT | Electronically recorded time taken (in hundredths of a second) for an animal to cover the reference distance (1.7 m) after leaving the weighing box at an average of 24 months of age. |
| Rectal Temperature (^o^C) | TEMP | Rectal temperature at approximately 27 months of age when ambient temperature was > 30°C |
| Faecal Egg Counts (eggs per gram) | EPG | Number of helminth eggs per gram of faeces recorded after weaning at an average of 10 months of age. |
| Penile Sheath Score | SHEATH | Subjective score for size of the abdominal skin extension of the penile sheath from 1 (large and pendulous) to 9 (close to the body, minimal skin) at an average of 18 months of age. For females this score corresponds to the extension of the navel. |
| Coat Colour | COLOUR | Subjective score for colour of white, cream, grey, red, tan, and black were transposed in that order to a quantitative scale from 1 (light) to 6 (dark). |
| Buffalo Fly Lesion Score | FLY | Subjective score for skin lesions caused by rubbing when challenged by *Haematobia irritans exigua* (buffalo fly) and *Stephanofilaria sp.* (nematode transmitted by the fly), recorded when flies were most prevalent and at an average of 5 years of age. Scores: 1 (no visible lesions), 2 (one lesion less than or equal to three inches), 3 (4 to 6 multiple lesions), 4 (7 to 10 multiple lesions up to at least three sites such as neck, belly and withers at 7 to 10 square inches in size), 5 (multiple lesions more extensive than score 4). |
| Tick Score | TICK | Scored as 0 (no ticks), 1 (10 or fewer ticks), 2 (11 to 30 ticks), 3 (31 to 80 ticks), 4 (81 to 150 ticks), and 5 (more than 150 ticks). Recorded when ticks were most prevalent and at an average of 24 months of age. |
| Coat Score | COAT | Recorded during post weaning cool months at < 12 months of age. Subjectively scored at 1/3rd score increments between 1 (extremely short and sleek coat) and 7 (very woolly coat) using the method of Turner (1960). Coat scores were converted to a continuous 21 point scale. |
| Body condition score | COND | Body condition visually assessed at an average of 30 months of age at the end of a growing (wet) season. Subjectively scored at 1/3rd score increments from 1 to 5, and subsequently converted to a continuous 15 point scale. |
| Yearling weight | YWT | Average of all live weights recorded between 300 and 420 days of age. |
