# Supplementary material for: The Genetic Architecture of Climatic Adaptation of Tropical Cattle
Source: PLoS One. 2014 Nov 24;9(11):e113284. doi: 10.1371/journal.pone.0113284 (PMC4242650; doi:10.1371/journal.pone.0113284)
Supplement: Table S2 — Bi-variate analysis for same trait in different sexes: Additive genetic variance (Va), heritability (h2) and genetic correlation (rg). (DOCX) [file pone.0113284.s005.docx]

Table S2. Bi-variate analysis for same trait in different sexes: Additive genetic variance (Va), heritability (h^2^) and genetic correlation (rg).

| Trait | Parameter | Brahman | | Tropical Composite | |
| --- | --- | --- | --- | --- | --- |
|  |  | **Males** | **Females** | **Males** | **Females** |
| FT | Va | 1595.5 | 715.6 | 877.7 | 552.9 |
|  | h^2^ | 0.536 | 0.390 | 0.496 | 0.430 |
|  | rg | 1.000 ± 0.000 | | 0.955 ± 0.078 | |
| TEMP | Va | 0.109 | 0.026 | 0.010 | 0.057 |
|  | h^2^ | 0.574 | 0.176 | 0.066 | 0.208 |
|  | rg | -0.249 ± 0.393 | | 1.000 ± 0.001 | |
| EPG^*^ | Va | -- | -- | 22250.3 | 44674.6 |
|  | h^2^ | -- | -- | 0.321 | 0.698 |
|  | rg | -- | | 1.000 ± 0.000 | |
| SHEATH | Va | 0.382 | 0.484 | 1.995 | 0.522 |
|  | h^2^ | 0.548 | 0.505 | 0.876 | 0.597 |
|  | rg | 0.949 ± 0.103 | | 0.893 ± 0.051 | |
| COLOUR | Va | 0.449 | 0.212 | 0.389 | 0.314 |
|  | h^2^ | 0.569 | 0.554 | 0.510 | 0.541 |
|  | rg | 1.000 ± 0.000 | | 1.000 ± 0.000 | |
| COAT | Va | 0.281 | 0.686 | 1.413 | 2.163 |
|  | h^2^ | 0.288 | 0.656 | 0.619 | 0.547 |
|  | rg | 0.860 ± 0.160 | | 0.925 ± 0.065 | |
| COND | Va | 0.075 | 0.370 | 0.080 | 0.263 |
|  | h^2^ | 0.444 | 0.568 | 0.405 | 0.473 |
|  | rg | 0.880 ± 0.117 | | 0.580 ± 0.123 | |
| YWT | Va | 303.10 | 162.83 | 481.76 | 247.05 |
|  | h^2^ | 0.602 | 0.457 | 0.673 | 0.534 |
|  | rg | 0.723 ± 0.117 | | 0.990 ± 0.049 | |

^*^EPG in Brahman was measured in females only.

NOTE: FLY and TICK were measured in females only.
