# Supplementary material for: The Genetic Architecture of Climatic Adaptation of Tropical Cattle
Source: PLoS One. 2014 Nov 24;9(11):e113284. doi: 10.1371/journal.pone.0113284 (PMC4242650; doi:10.1371/journal.pone.0113284)
Supplement: Table S3 — Number of significant SNP from GWAS at different levels of stringency for each trait and false discovery rate between brackets. (DOCX) [file pone.0113284.s006.docx]

Table S3. Number of significant SNP from GWAS at different levels of stringency for each trait and false discovery rate between brackets.

| Trait | BRAHMAN | | | | TROPICAL COMPOSITE | | | | BOTH BREEDS | |
| --- | --- | --- | --- | --- | --- | --- | --- | --- | --- | --- |
|  | **<0.001** | | **<0.0001** | | **<0.001** | | **<0.0001** | | **<0.001** | **<0.0001** |
| **FT** | 1870 | (0.39) | 253 | (0.29) | 3480 | (0.21) | 581 | (0.13) | 15 | 0 |
| **TEMP** | 1086 | (0.67) | 141 | (0.52) | 856 | (0.85) | 69 | (1.06) | 2 | 0 |
| **EPG** | 1772 | (0.41) | 239 | (0.30) | 2461 | (0.30) | 479 | (0.15) | 8 | 0 |
| **SHEATH** | 5766 | (0.13) | 2008 | (0.04) | 14269 | (0.05) | 10339 | (0.01) | 2122 | 1401 |
| **COLOUR** | 6154 | (0.12) | 2880 | (0.03) | 9206 | (0.08) | 6029 | (0.01) | 71 | 11 |
| **FLY** | 1851 | (0.39) | 309 | (0.24) | 1942 | (0.37) | 296 | (0.25) | 2 | 0 |
| **TICK** | 1045 | (0.70) | 154 | (0.47) | 1417 | (0.51) | 133 | (0.55) | 1 | 0 |
| **COAT** | 3026 | (0.24) | 739 | (0.10) | 8073 | (0.09) | 3046 | (0.02) | 61 | 14 |
| **COND** | 4683 | (0.15) | 1711 | (0.04) | 7371 | (0.10) | 2334 | (0.03) | 463 | 214 |
| **YWT** | 3751 | (0.19) | 1010 | (0.07) | 14000 | (0.05) | 7302 | (0.01) | 551 | 167 |
| **Average** | 3100 | (0.23) | 944 | (0.08) | 6308 | (0.11) | 3061 | (0.02) | 330 | 181 |
