# Supplementary material for: The Genetic Architecture of Climatic Adaptation of Tropical Cattle
Source: PLoS One. 2014 Nov 24;9(11):e113284. doi: 10.1371/journal.pone.0113284 (PMC4242650; doi:10.1371/journal.pone.0113284)
Supplement: Table S4 — Summary table of SNP association: overlap regions between Brahman and Tropical Composite (P<0.0001 both breeds). (DOCX) [file pone.0113284.s007.docx]

Table S4. Summary table of SNP association: overlap regions between Brahman and Tropical Composite (P<0.0001 both breeds).

| **Trait** | **BTA** | **Mb** | **Genes (<=3Kb from SNP)** |
| --- | --- | --- | --- |
| **SHEATH** | 5 | 28 | ANKRD33, GALNT6 |
|  | 5 | 31 | ADCY6, C5H12orf41, CACNB3, DDX23, LALBA, LOC786658 |
|  | 5 | 33 | FAM113B |
|  | 5 | 36 | IRAK4, TMEM117 |
|  | 5 | 38 | PPHLN1 |
|  | 5 | 39 | PDZRN4 |
|  | 5 | 40 | CNTN1, LRRK2 |
|  | 5 | 41 | KIF21A |
|  | 5 | 42 | CPNE8, KIF21A, PTPRR |
|  | 5 | 43 | KCNMB4, LOC531070, MGC139000, PTPRB, PTPRR, RAB3IP |
|  | 5 | 44 | LOC531070 |
|  | 5 | 45 | CPD, IL22, IL26, RAP1B |
|  | 5 | 46 | CAND1, LOC514916 |
|  | 5 | 47 | GRIP1, HELB, IRAK3, TMBIM4 |
|  | 5 | 48 | LEMD3, MSRB3, WIF1 |
|  | 5 | 49 | GNS, LOC783207, LOC783553, RASSF3, SRGAP1, TBC1D30, TBK1, WIF1, XPOT |
|  | 5 | 50 | PPM1H, SRGAP1 |
|  | 5 | 51 | PPM1H |
|  | 5 | 52 | LOC617908 |
|  | 5 | 53 | SLC16A7 |
|  | 5 | 54 | LRIG3, SLC16A7 |
|  | 5 | 55 | LOC514014 |
|  | 5 | 56 | ARHGAP9, B4GALNT1, CYP27B1, DCTN2, DDIT3, FAM119B, GGAP2, GLI1, INHBE, KIF5A, LOC533894, MARCH9, METTL1, MIR2431, MYO1A, NAB2, PIP4K2C, R3HDM2, SDR9C7, SLC26A10, STAC3, STAT6, TAC3, TMEM194A, TSFM, TSPAN31, |
|  | 5 | 57 | BAZ2A, GLS2, HSD17B6, MGC142702, MIP, PTGES3, RBMS2, SPRYD4, TIMELESS |
|  | 5 | 58 | LOC618816 |
|  | 5 | 60 | ELK3, HAL, LOC538993, LOC788615, LOC789016, LTA4H, NEUROD4, NTN4, PCTK2 |
|  | 5 | 61 | LOC787705, PCTK2 |
|  | 5 | 63 | LOC516896 |
|  | 5 | 64 | LOC516896, NR1H4, UHRF1BP1L |
|  | 5 | 65 | ANO4, CHPT1, LOC510487, MYBPC1, SLC5A8, SYCP3 |
|  | 5 | 66 | CCDC53, DRAM1, IGF1, LOC100139879, LOC615510, PAH |
|  | 5 | 69 | APPL2 |
| **COLOUR** | 13 | 63 | BPIFB9 |
|  | 13 | 64 | ASIP |
|  | 13 | 65 | RBM39 |
| **COAT** | 8 | 36 | R-PTP-delta |
|  | 8 | 39 | JAK2, RCL1 |
|  | 8 | 40 | LOC531778 |
| **COND** | 14 | 24 | LYN, MOS, RPS20, TGS1, TMEM68, XKR4 |
|  | 14 | 25 | CHCHD7, IMPAD1, LOC526726, PLAG1 |
|  | 14 | 26 | FAM110B, LOC510507, LOC530316, TOX |
|  | 14 | 27 | CA8, CHD7 |
|  | 15 | 66 | CD44 |
| **YWT** | 6 | 36 | SNCA |
|  | 6 | 37 | HERC3 |
|  | 6 | 38 | LAP3, LOC540095 |
|  | 14 | 24 | LYN, MOS, TMEM68, XKR4 |
|  | 14 | 25 | CHCHD7, IMPAD1, LOC526726, PENK, PLAG1, SDR16C6 |
|  | 14 | 26 | TOX |
|  | 14 | 27 | CA8 |
|  | 14 | 28 | ASPH, CHD7, RLBP1L1 |
|  | 14 | 29 | GGH, TTPA, YTHDF3 |
|  | 30 | 19 | MAP7D3, SLC9A6 |
