# Supplementary material for: The Genetic Architecture of Climatic Adaptation of Tropical Cattle
Source: PLoS One. 2014 Nov 24;9(11):e113284. doi: 10.1371/journal.pone.0113284 (PMC4242650; doi:10.1371/journal.pone.0113284)
Supplement: Table S5 — Selected Pleiotropy results: Studentized SNP effects across the 10 phenotypes for candidate SNP and its closest gene. Highlighted in yellow are effects> 2.0 in absolute value. (DOCX) [file pone.0113284.s008.docx]

Table S5. Selected Pleiotropy results: Studentized SNP effects across the 10 phenotypes for candidate SNP and its closest gene. Highlighted in yellow are effects > 2.0 in absolute value.

|  | FT | TEMP | EPG | SHEATH | COLOUR | FLY | TICK | COAT | COND | YWT |
| --- | --- | --- | --- | --- | --- | --- | --- | --- | --- | --- |
| **BovineHD1400007257 BTA14:25009960 PLAG1** | | | | | | | | | | |
| BRM | 0.000 | 0.000 | -2.878 | -0.690 | 0.692 | -0.148 | -0.793 | -4.686 | 8.195 | -5.778 |
| COMP | 0.785 | -0.206 | -0.253 | 1.165 | 0.721 | -2.389 | 0.680 | 0.002 | 6.303 | -4.999 |
| **BovineHD1400008334 BTA14:28799088 ASPH** | | | | | | | | | | |
| BRM | -0.410 | -0.659 | -2.383 | 0.139 | 0.653 | -1.623 | -0.470 | -4.423 | 6.991 | -4.538 |
| COMP | -0.123 | -0.700 | 0.617 | -0.930 | 0.209 | -1.619 | -0.561 | -0.832 | 2.645 | -5.849 |
| **BovineHD1400006535 BTA14:22680469 PCMTD1** | | | | | | | | | | |
| BRM | -0.655 | -0.413 | 2.640 | -0.478 | -1.012 | 0.765 | -0.612 | 2.231 | -5.239 | 2.432 |
| COMP | -1.227 | 0.460 | -0.343 | -0.559 | -0.675 | 1.345 | -0.636 | 0.504 | -4.166 | 0.006 |
| **BovineHD0500013895 BTA5:48069099 HMGA2** | | | | | | | | | | |
| BRM | -1.007 | 0.133 | 0.587 | -10.361 | -0.286 | -0.359 | 0.910 | 1.780 | -3.964 | 0.018 |
| COMP | 0.886 | 0.481 | -1.617 | -41.397 | 6.291 | 4.192 | 2.046 | -0.914 | -5.244 | 8.146 |
| **BovineHD0500016019 BTA5:56371072 INHBC** | | | | | | | | | | |
| BRM | 0.648 | 1.753 | 0.429 | -8.204 | 1.044 | -1.300 | 0.426 | 1.515 | -3.485 | -0.400 |
| COMP | 0.057 | -0.359 | -2.869 | -26.961 | 10.142 | 3.025 | 0.034 | 0.330 | -4.965 | 4.173 |
| **BovineHD0500012231 BTA5:42818597 PTPRR** | | | | | | | | | | |
| BRM | 0.249 | -0.862 | -0.914 | -5.511 | -0.511 | -0.151 | 0.235 | 3.894 | -1.273 | 0.409 |
| COMP | -0.592 | -0.472 | -0.632 | -18.522 | 5.753 | 1.471 | 0.271 | -2.063 | -3.827 | 3.282 |
| **BovineHD0600005016 BTA6:18348426 LEF1** | | | | | | | | | | |
| BRM | 0.680 | -0.167 | 0.731 | 0.662 | -20.531 | 0.388 | 0.352 | 0.228 | -0.735 | 0.083 |
| COMP | -0.606 | 0.304 | -0.817 | 2.479 | -2.741 | 0.915 | -2.760 | -0.915 | 0.002 | -4.335 |
| **BovineHD0600011548 BTA6:42442392 KCNIP4** | | | | | | | | | | |
| BRM | 0.382 | -0.660 | -0.726 | -1.510 | 0.307 | -1.485 | 0.000 | -0.825 | -4.680 | 3.587 |
| COMP | 1.463 | -2.150 | 0.393 | -0.036 | -0.042 | 0.507 | 0.720 | -0.423 | -3.411 | 8.102 |
| **BovineHD1300018328 BTA13:64228423 SNORA73** | | | | | | | | | | |
| BRM | -0.096 | 1.169 | -0.143 | -0.139 | 11.329 | -0.665 | -3.553 | -0.653 | 0.199 | -0.767 |
| COMP | 0.823 | 1.215 | -0.816 | -0.647 | 4.156 | 0.260 | -0.281 | 0.797 | -3.711 | -0.529 |
| **BovineHD0800011904 BTA8:40191727 SLC1A1** | | | | | | | | | | |
| BRM | 1.017 | -0.681 | -0.006 | -3.491 | -0.677 | -0.227 | 2.809 | 6.180 | 1.290 | -0.474 |
| COMP | 0.547 | 0.295 | -1.501 | -1.625 | -0.307 | -0.020 | 0.711 | 4.064 | -3.116 | 0.961 |
| **BovineHD2000004271 BTA20:13306855 SREK1** | | | | | | | | | | |
| BRM | -0.001 | 2.118 | 0.403 | 0.150 | -0.747 | 6.004 | -0.588 | 0.038 | -0.422 | 0.152 |
| COMP | -1.607 | 1.265 | 2.203 | 0.138 | 3.004 | -2.171 | -0.366 | -4.164 | 0.266 | -1.292 |
| **BovineHD2000008759 BTA20:29858632 MRPS30** | | | | | | | | | | |
| BRM | -1.943 | 1.919 | 0.914 | -0.836 | 0.868 | 1.984 | 0.694 | 1.502 | 0.838 | -3.941 |
| COMP | -0.566 | 0.000 | 1.227 | 2.213 | -0.951 | -4.736 | -0.005 | 10.736 | 0.618 | -0.004 |
| **BovineHD0200021209 BTA2:74082443 TSN** | | | | | | | | | | |
| BRM | -3.319 | 0.686 | 0.500 | -1.193 | -0.257 | -1.250 | -0.536 | 0.037 | -3.428 | 2.188 |
| COMP | -4.734 | 1.247 | 0.438 | -0.681 | -0.167 | -0.356 | 2.494 | 0.658 | -1.325 | 0.216 |
| **BovineHD3000006012 BTAX:18110348 HPRT1** | | | | | | | | | | |
| BRM | -2.256 | -0.544 | 0.216 | 0.412 | 0.715 | -0.363 | 0.784 | -1.314 | 0.570 | -5.580 |
| COMP | 0.584 | 0.741 | -1.499 | -0.758 | 0.108 | -2.722 | 0.047 | -3.608 | -0.594 | -1.349 |
| **BovineHD0400022663 BTA4:82174354 POUF2** | | | | | | | | | | |
| BRM | 1.018 | 0.519 | 0.650 | -0.645 | 0.267 | 1.097 | -0.046 | -0.045 | 5.155 | 3.049 |
| COMP | -0.434 | -0.581 | -0.883 | -0.140 | -0.363 | -3.664 | 0.714 | 0.665 | 3.737 | -1.771 |
| **Hapmap57082-ss46526507 BTA15:66540919 CD44** | | | | | | | | | | |
| BRM | -0.387 | -0.727 | -3.114 | -0.124 | -0.743 | -0.036 | 0.914 | -0.498 | -4.503 | -0.412 |
| COMP | -0.595 | 0.456 | 0.835 | -0.191 | 0.071 | -2.127 | 2.968 | -0.768 | 4.572 | 0.653 |
| **BovineHD1500022318 BTA15:76506367 CHST1** | | | | | | | | | | |
| BRM | -0.792 | 0.334 | 0.827 | -0.493 | 0.963 | -3.415 | -0.021 | -0.633 | -3.745 | -2.066 |
| COMP | -0.402 | -0.122 | -0.409 | 0.296 | 0.804 | 1.074 | -0.229 | 5.588 | 0.000 | 0.833 |
