# Supplementary material for: The Genetic Architecture of Climatic Adaptation of Tropical Cattle
Source: PLoS One. 2014 Nov 24;9(11):e113284. doi: 10.1371/journal.pone.0113284 (PMC4242650; doi:10.1371/journal.pone.0113284)
Supplement: Table S6 — Correlation between SNP additive effects (n = 729,068), and statistical model R2 estimated in the Brahman population with and without fitting the covariate “% indicine” in the model. (DOCX) [file pone.0113284.s009.docx]

**Table S6. Correlation between SNP additive effects (n=729,068), and statistical model R^2^ estimated in the Brahman population with and without fitting the covariate “% indicine” in the model.**

| **Trait** | **Parameter** | **SNP additive effect** | | **statistical model R^2^** | |
| --- | --- | --- | --- | --- | --- |
|  |  | **Without percent indicine** | **With percent indicine** | **Without percent indicine** | **With percent indicine** |
| **COAT** | Mean | -0.0027 | -0.0003 | 54.95 | 55.08 |
|  | Std | 0.1088 | 0.1076 |  |  |
|  | Min | -3.924 | -3.981 |  |  |
|  | Max | 3.055 | 3.052 |  |  |
|  | Correlation | 0.9814396 | |  |  |
| **COLOUR** | Mean | -0.0033 | 0.0002 | 13.45 | 16.39 |
|  | Std | 0.0833 | 0.0802 |  |  |
|  | Min | -2.838 | -2.797 |  |  |
|  | Max | 2.597 | 2.557 |  |  |
|  | Correlation | 0.9248053 | |  |  |
| **COND** | Mean | 0.0011 | -0.0002 | 61.42 | 61.57 |
|  | Std | 0.0746 | 0.0753 |  |  |
|  | Min | -2.286 | -2.254 |  |  |
|  | Max | 2.411 | 2.366 |  |  |
|  | Correlation | 0.9871247 | |  |  |
| **EPG** | Mean | 0.6237 | -0.1012 | 31.33 | 31.34 |
|  | Std | 42.8454 | 29.7659 |  |  |
|  | Min | -1199.45 | -1200.98 |  |  |
|  | Max | 769.07 | 771.122 |  |  |
|  | Correlation | 0.9868908 | |  |  |
| **FLY** | Mean | 0.0018 | 0.0000 | 18.67 | 18.93 |
|  | Std | 0.0894 | 0.09 |  |  |
|  | Min | -3.075 | -3.054 |  |  |
|  | Max | 3.771 | 3.793 |  |  |
|  | Correlation | 0.9845703 | |  |  |
| **FT** | Mean | 0.0553 | 0.0093 | 42.19 | 42.24 |
|  | Std | 4.7058 | 4.7158 |  |  |
|  | Min | -188.424 | -188.667 |  |  |
|  | Max | 188.424 | 188.667 |  |  |
|  | Correlation | 0.9960248 | |  |  |
| **SHEATH** | Mean | -0.0027 | 0.0001 | 43.94 | 44.53 |
|  | Std | 0.1032 | 0.1015 |  |  |
|  | Min | -3.021 | -2.957 |  |  |
|  | Max | 2.671 | 2.605 |  |  |
|  | Correlation | 0.9708901 | |  |  |
| **TEMP** | Mean | 0.0001 | 0.0000 | 59.01 | 59.01 |
|  | Std | 0.0481 | 0.0484 |  |  |
|  | Min | -1.826 | -1.825 |  |  |
|  | Max | 1.474 | 1.475 |  |  |
|  | Correlation | 0.9996377 | |  |  |
| **TICK** | Mean | -0.001 | 0.0033 | 55.87 | 56.69 |
|  | Std | 0.1099 | 0.1746 |  |  |
|  | Min | -2.804 | -2.800 |  |  |
|  | Max | 2.804 | 2.800 |  |  |
|  | Correlation | 0.8665777 | |  |  |
| **YWT** | Mean | -0.0262 | 0.0041 | 67.54 | 67.57 |
|  | Std | 2.1245 | 2.1292 |  |  |
|  | Min | -67.152 | -67.158 |  |  |
|  | Max | 80.206 | 80.871 |  |  |
|  | Correlation | 0.9917861 | |  |  |
